# Improving Brain Metabolite Detection with a Combined Low-Rank Approximation and Denoising Diffusion Probabilistic Model Approach

**DOI:** 10.3390/bioengineering11111170

**Published:** 2024-11-20

**Authors:** Yeong-Jae Jeon, Kyung Min Nam, Shin-Eui Park, Hyeon-Man Baek

**Affiliations:** 1 Department of Health Sciences and Technology, Gachon Advanced Institute for Health Sciences and Technology, Gachon University, Incheon 21999, Republic of Korea; yeong@gachon.ac.kr; 2 High Field MR Research Group, Center for Image Sciences, University Medical Centre Utrecht, Heidelberglaan 100, P.O. Box 85500, 3584 CX Utrecht, The Netherlands; K.M.Nam@umcutrecht.nl; 3 Department of Biomedical Science, Lee Gil Ya Cancer and Diabetes Institute, Gachon University, Incheon 21999, Republic of Korea; shineuipark@gmail.com; 4 Department of Molecular Medicine, Lee Gil Ya Cancer and Diabetes Institute, Gachon University, Incheon 21999, Republic of Korea

**Keywords:** ^1^H MRS, denoising, functional MRS, low-rank approximation, CSVD, pain, anterior cingulate cortex (ACC), denoising diffusion probabilistic model (DDPM)

## Abstract

In vivo proton magnetic resonance spectroscopy (MRS) is a noninvasive technique for monitoring brain metabolites. However, it is challenged by a low signal-to-noise ratio (SNR), often necessitating extended scan times to compensate. One of the conventional techniques for noise reduction is signal averaging, which is inherently time-consuming and can lead to participant discomfort, thus posing limitations in clinical settings. This study aimed to develop a hybrid denoising strategy that integrates low-rank approximation and denoising diffusion probabilistic model (DDPM) to enhance MRS data quality and shorten scan times. Using publicly available ^1^H MRS datasets from 15 subjects, we applied the Casorati SVD and DDPM to obtain baseline and functional data during a pain stimulation task. This method significantly improved SNR, resulting in outcomes comparable to or better than averaging over 32 signals. It also provided the most consistent metabolite measurements and adequately tracked temporal changes in glutamate levels, correlating with pain intensity ratings after heating. These findings demonstrate that our approach enhances MRS data quality, offering a more efficient alternative to conventional methods and expanding the potential for the real-time monitoring of neurochemical changes. This contribution has the potential to advance MRS techniques by integrating advanced denoising methods to increase the acquisition speed and enhance the precision of brain metabolite analyses.

## 1. Introduction

In vivo proton magnetic resonance spectroscopy (MRS) is a noninvasive tool used to detect and monitor abnormal metabolism in the concentration of various metabolites in the brain from their normal levels [1]. Functional MRS, which involves collecting MRS data in series over time, has been employed to monitor the dynamic changes in metabolite levels in response to various stimuli [2,3,4,5]. The prominent metabolites identified were N-acetylaspartate (NAA), total creatine (PCr+Cr or tCr), total choline (tCho), Glx (sum of glutamate and glutamine), and myo-inositol (m-Ins).

However, its practical utility is limited by a low signal-to-noise ratio (SNR), mainly due to the low concentrations of metabolites, which makes further analysis challenging [6]. The traditional method for reducing noise is to average multiple noisy MRS acquisitions at the same location. However, this approach requires many repetitions, and the prolonged total acquisition time can be problematic for patient comfort and compromises time resolution in functional MRS. For example, at 3T, a typical MRS scan with 32 signal averages (NSA) takes 3 min and 12 s, whereas functional MRS, with 10 spectra of 16 NSA each, extends the scan time to 22 min and 4 s [2]. Therefore, to reduce participant discomfort and effectively observe functional neurochemical changes, it is desirable to obtain fast MRS data while maintaining an adequate SNR.

Denoising is a potentially useful approach to enhance the SNR and reduce scan time, and many methods have made significant advancements in recent years [7,8,9,10,11,12,13,14]. Among these, the low-rank approximation approach utilizing the spatial or temporal dependence of data has been demonstrated to be particularly effective for denoising MRS data [7,9,11,12]. However, these methods may struggle with in vivo data owing to deviations from simulated data under ideal conditions and often do not leverage previously acquired spectra.

Many deep learning (DL) methods have been explored for the MRS denoising problem [10,13,14]. However, training DL models typically requires a large quantity of low-noise reference data that is not always available in practice [10]. Consequently, simulated data are often used to train these models. However, ensuring that the models perform well on real data, not just simulations, can be challenging [13]. Moreover, while convolutional neural network (CNN)-based supervised learning approaches reduce noise outside the training range, they may result in substantial estimation bias [14]. Recent studies on MRSI [13,15,16] have shown that CNN architectures, such as deep complex convolutional autoencoders (DCCAE), can achieve excellent denoising performance for MRSI data. Implementing effective CNN-based denoising, however, typically demands large training datasets with ground truth, advanced MRS simulations, complex convolutional operations, and sophisticated optimization techniques. Using an insufficiently trained model (e.g., a pretrained model) without these adjustments often leads to suboptimal results, as demonstrated in Supplementary Materials Figure S8. To address similar challenges, alternative unsupervised methods [17,18,19,20,21] such as diffusion probabilistic models (DDPMs) [17] have gained attention in medical image analyses [18]. DDPMs have been successfully applied to denoise OCT images [19], PET scans [20], and diffusion MRI [21], achieving state-of-the-art results by learning noise distributions and outperforming traditional methods in terms of noise reduction and detail preservation. The application of DDPMs involves a probabilistic process that iteratively removes noise from a noisy input, gradually reconstructing a clearer version of the data. DDPMs operate through two primary phases. The diffusion process and the reverse denoising process. In the forward diffusion process, the model gradually adds noise to a clean input signal over several steps, ultimately transforming it into an almost random noise distribution. Each step in this process is defined predictably, allowing for the model to learn how noise is incrementally added. In the reverse denoising process, the model is trained to gradually remove noise step-by-step, reconstructing the original signal from highly noisy data. This denoising process starts from the final (or sometimes intermediate) noisy state and moves backward, with each step reducing noise and moving closer to the clean signal. By learning the probability distributions of data and noise at each stage, the model becomes adept at reconstructing clear signals from noisy inputs.

Building on insights from previous research, this study focused on applying both low-rank approximation-based and DDPM-based denoising techniques to MRS data. We begin by applying each method individually to evaluate its performance and then explore the potential benefits of combining these approaches. Our primary objective was to determine whether denoising could sufficiently enhance the data quality, potentially reducing the need for signal averaging. Furthermore, we will extend our investigation to functional MRS datasets to assess the ability of these methods to monitor metabolite changes accurately over time.

## 2. Materials and Methods

### 2.1. Datasets

We used publicly available ^1^H MRS datasets [2] acquired from 15 subjects using a 3 T Philips Achieva scanner (Best, The Netherlands) with a single-channel transmit–receive (T/R) head coil. MRS data were collected from the anterior cingulate cortex (ACC) at a resolution of $30\times 25\times 15\;mm^{3}$. Both baseline and functional datasets are provided in two formats as a vendor proprietary raw data format: ‘spar/sdat’ and ‘list/data’. The ‘spar/sdat’ format contained signal-averaged data, while the ‘list/data’ format contained raw, non-averaged data. All scans were conducted in accordance with ethical review board guidelines, and informed consent was obtained from all participants [2].

#### 2.1.1. Baseline MRS Dataset

Baseline MRS data were acquired using a PRESS sequence with acquisition parameters of TR/TE = 4000/22 ms. Each subject provided 32 non-averaged (‘list/data’) and averaged (‘spar/sdat’) spectra, with a scan time of 3 min and 12 s, including 16 spectra that were non-water-suppressed. The term ‘baseline’ refers to data collected during a resting state without external stimuli.

#### 2.1.2. Functional MRS Dataset

**Figure 1:**
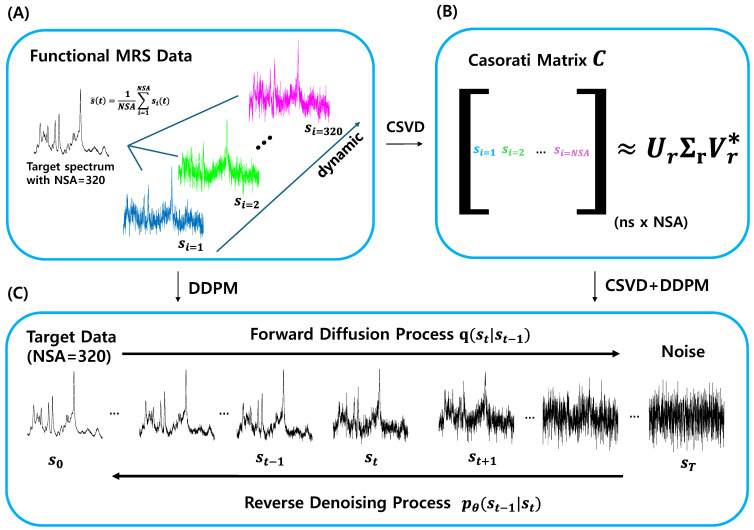
Overview of the CSVD and DDPM denoising process for functional MRS data. (**A**) The functional MRS data are displayed, illustrating the target spectrum with different signals from 1 to 320. (**B**) Casorati matrix C is constructed using the functional MRS dataset and decomposed by the Casorati singular value decomposition (CSVD) into components $U_{r}\sum_{r}V_{r}^{*}$. (**C**) Denoising diffusion probabilistic model (DDPM) is trained to the target data, illustrating the forward diffusion process from the initial state $s_{0}$ to noise $s_{T}$ and the reverse denoising process to recover the signal. NSA represents the number of signal averages; subscript t in DDPM indicates diffusion steps, while subscript *i* denotes the index for *i*-th non-averaged MRS data; and ns refers to the number of sample points.

Functional MRS data were also acquired using a PRESS sequence with the same parameters of TR/TE. This dataset comprised 320 non-averaged spectra (‘list/data’, including water) from each subject (Figure 1A), with a total scan time of 22 min and 4 s. The ‘functional’ dataset was obtained during the application of external stimuli, specifically a pain task involving the application of capsaicin, followed by heat (~41 °C) to the forearm for a period of 4.4 min, starting 9 min into the scan. Detailed experimental information can be found in [2].

### 2.2. Denoising Methods

#### 2.2.1. Casorati Singular Value Decomposition (CSVD)

Using the non-averaged MRS data provided in the ‘list/data’ format, a Casorati matrix C can be constructed (Figure 1B). In this matrix, the rows correspond to the number of data points and the columns correspond to the number of signals. Owing to the strong correlations between the columns, C is typically a low-rank matrix [7,9,12]. Singular value decomposition (SVD) was applied to approximate C using rank-r approximation:

(1) $$C\approx U_{r}\Sigma_{r}V_{r}^{*}$$

where $U_{r}$ is the matrix consisting of the first r left singular vectors of C, $\Sigma_{r}$ is the diagonal matrix containing the top *r* singular values of C, and $V_{r}^{*}$ is the matrix formed by taking the first *r* rows of the conjugate transpose of V. To obtain the denoised spectrum $\hat{s}$ from the noisy spectrum s, we projected s onto the subspace spanned by $U_{r}$. This problem can be formulated as

(2) $$\arg\underset{\hat{s}}{\min}{\left\|\hat{s}\;-s\right\|}_{2}^{2}+\lambda {\left\|U_{r}U_{r}^{*}\hat{s}\;-s\right\|}_{2}^{2}$$

where λ is a regularization parameter. The closed-form solution to this problem is given by

(3) $$\hat{s}=(I+\lambda {\left(U_{r}U_{r}^{*}-I\right)}^{*}{\left(U_{r}U_{r}^{*}-I\right))}^{-1}s$$

where I denotes the identity matrix. The derivation of the closed-form solution is provided in Appendix A.1.

#### 2.2.2. Denoising Diffusion Probabilistic Model (DDPM)

We used the averaged signal data as a high SNR target for clean reference spectra and used it for diffusion model training (Figure 1C). As the diffusion probabilistic model aims to learn the noise pattern instead of the signal [19], the average output $s_{0}$ can still be used as a clean spectrum for training purposes. Figure 1C shows the Markov chain in the forward and reverse directions.

(4) $$q\left(s_{1:T}|s_{0}\right)=\prod_{t=1}^{T}q\left(s_{t}|s_{t-1}\right)\;\;\;\;\;\;\;\;\;\;$$

(5) $$p_{\theta }(s_{0:T})=p(s_{T})\prod_{t=1}^{T}p_{\theta }(s_{t-1}|s_{t})$$

where $q(s_{0})$ is the data distribution, and $p\left(s_{T}\right)=N(s_{T};0,\;I)$. Here, θ represents the model parameters. Our goal is to train a deep model $p_{\theta }$ to reconstruct a noisy spectrum s, using an adjustable parameter *t*, where higher values of *t* indicate more denoising steps.

In the forward process, we add small Gaussian noise with a variance schedule $\left\{\beta_{1},\ldots ,\beta_{T}\right\}$, where $\beta_{t}\in \left(0,\;1\right)$:

(6) $$q\left(s_{t}|s_{t-1}\right)=N(s_{t};\sqrt{\alpha_{t}}s_{t-1},\;\beta_{t}I)$$

where $\alpha_{t}=1-\beta_{t}$. The distribution $q\left(s_{t}|s_{0}\right)$ is given by

(7) $$q\left(s_{t}|s_{0}\right)=N(s_{t};\sqrt{{\bar{\alpha }}_{t}}s_{0},\;\left(1-{\bar{\alpha }}_{t}\right)I)$$

where ${\bar{\alpha }}_{t}=\prod_{l=1}^{t}\alpha_{l}$. The sampling of $s_{t}$ can be reparametrized as

(8) $$s_{t}=\sqrt{{\bar{\alpha }}_{t}}s_{0}+\sqrt{1-{\bar{\alpha }}_{t}}\epsilon$$

where ϵ ~ N(0, I). In the reverse process, the transition is also modeled as a Gaussian distribution:

(9) $$p_{\theta }\left(s_{t-1}|s_{t}\right)=N(s_{t-1};\mu_{\theta }\left(s_{t},t\right),\;\Sigma_{\theta }\left(s_{t},t\right))$$

We set the variance $\Sigma_{\theta }\left(s_{t},t\right)=\beta_{t}$ and learn to predict the mean $\mu_{\theta }$. The loss function aims to minimize the KL divergence between the forward and reverse distributions.

(10) $$\begin{array}{c} L=D_{KL}(\left.q\left(s_{T}|s_{0}\right)\;\right|\left|\;p\left(s_{T}\right)\;)\;\right.\\ \;\;\;\;\;\;+\sum_{t=2}^{T}D_{KL}(\left.q\left(s_{t-1}|s_{t},s_{0}\right)\;\right|\left|\;p_{\theta }\left(s_{t-1}|s_{t}\right))\right.-\log(p_{\theta }\left(s_{0}|s_{1}\right)) \end{array}$$

With fixed variance schedule, the first term is constant, and the third term is negligible. Thus, we focused on minimizing the second loss term $L_{t-1}$. Given Equations (7) and (9), $L_{t-1}$ is the KL divergence of two Gaussian distributions, the loss function can be reduced to Equation (11).

(11) $$L_{t-1}=\frac{1}{{2\beta }_{t}}{\left\|{\tilde{\mu }}_{t}\left(s_{t},s_{0}\right)-\mu_{\theta }\left(s_{t},t\right)\right\|}^{2}$$

where

(12) $${\tilde{\mu }}_{t}\left(s_{t},s_{0}\right)=\frac{\sqrt{{\bar{\alpha }}_{t-1}}\beta_{t}}{1-{\bar{\alpha }}_{t}}s_{0}+\frac{\sqrt{\alpha_{t}}\left(1-{\bar{\alpha }}_{t-1}\right)}{1-{\bar{\alpha }}_{t}}s_{t}$$

(13) $$\mu_{\theta }\left(s_{t},\;t\right)=\frac{1}{\sqrt{\alpha_{t}}}\left(s_{t}-\frac{\beta_{t}}{\sqrt{1-{\bar{\alpha }}_{t}}}\epsilon_{\theta }(s_{t},t)\;\right)$$

Thus, to minimize $L_{t-1}$, we can set the mean prediction $\mu_{\theta }$ equal to ${\tilde{\mu }}_{t}$, the model learns to approximate the Gaussian noise ϵ with a neural network $\epsilon_{\theta }(s_{t},t)$ instead of directly approximating ${\tilde{\mu }}_{t}$. A detailed derivation of the above equations is provided in References [17,18,19,20,21].

#### 2.2.3. Implementation Details

For CSVD denoising, we processed the data using custom in-house programs developed with MATLAB (R2024a) running on a PC equipped with a 3.61-GHz Intel Core i7 processor and 32 GB of RAM. The optimal rank threshold r and regularization parameter λ were determined as follows: for the baseline MRS data shown in Figures S1–S3, r was set to 2 and λ to 500; for the functional MRS data in Figure S3, r was set to 5 and λ to 100. These parameter choices were made based on the combinations that achieved the highest PSNR in each dataset.

To implement DDPM denoising, we utilized the MATLAB example script ‘GenerateImagesUsingDiffusionExample.mlx’ (https://www.mathworks.com/help/deeplearning/ug/generate-images-using-diffusion.html (accessed on 7 March 2024)). To train the DDPM model, we generated a training dataset using the functional MRS data from 10 subjects. The NSA320 averaged spectra, without water and spurious signals, were zero-padded from 2048 to 4096 data points and then randomly zero-order phase-modulated within 180-degree range. The resulting 1D data (i.e., 4096 × 1) were reshaped into a 2D matrix (64 × 64), and the intensity was normalized to the range [−1, 1] for use as the network input. A total of 20,000 training samples were generated, with 2000 samples from each of the 10 subjects. The baseline MRS dataset was used as the test set. The variance schedule is set to increase from 1 × 10^−5^ to 1 × 10^−3^ over T = 1000 steps. The network architecture was based on the model used in [17] and was trained on an NVIDIA GeForce RTX 3070 8 GB GPU for 11 epochs, with a batch size of 1, using the Adam optimizer. The learning rate was 0.0005, with a gradient decay factor of 0.9 and a squared gradient decay factor of 0.9999. Training was stopped early because of the lack of further reduction in loss (Figure S9). The entire training process took approximately 50 h. For DDPM denoising, the reverse denoising steps were examined at 1, 2, 3, 4, 5, 6, 7, 10, 20, 50, and 100 for the DDPM-only model and the combined model (Figures S5 and S7). The reverse denoising steps were set to ten for the DDPM-only model due to quantification errors for functional data (Figure S10) and two for the combined CSVD+DDPM model (Figures S6 and S7C,D).

### 2.3. Data Analysis

To evaluate the effectiveness of the denoising methods, the baseline data were visually inspected. Subsequently, we utilized LCModel analysis [22] to assess the performance of various methods for producing high-quality MR spectra. This analysis was conducted on all individual non-averaged spectra for each method, including both the baseline (N_spect_ = 15×32; number of subjects × number of spectra) and functional data (N_spect_ = 15×320). We compared the signal enhancements by analyzing the SNR values and investigated the line-broadening effects using full-width at half-maximum (FWHM) measurements. Moreover, we assessed the quantification uncertainty of each method using Cramer–Rao lower bound (CRLB) values and compared the metabolite concentration changes. All denoising methods were applied to non-averaged NSA1 data, with NSA32 serving as reference data. Statistical comparisons were performed using pairwise Student’s *t*-tests. We also applied these methods to functional MRS data to observe the temporal changes in key metabolites for each denoising approach. Finally, we investigated changes in glutamate levels in several notable individuals. To investigate temporal metabolite-level changes, a moving window average was applied with a window size of 16. The Glu changes in individual subjects are presented in Figure S11. We excluded two data (subjects #14 and #15 in Figure S11) from the group analysis because of identified artifacts (red arrows in Figure S11). We analyzed the variations in key metabolites and assessed the correlation with pain intensity ratings.

## 3. Results

### 3.1. Baseline MRS Dataset

**Figure 2:**
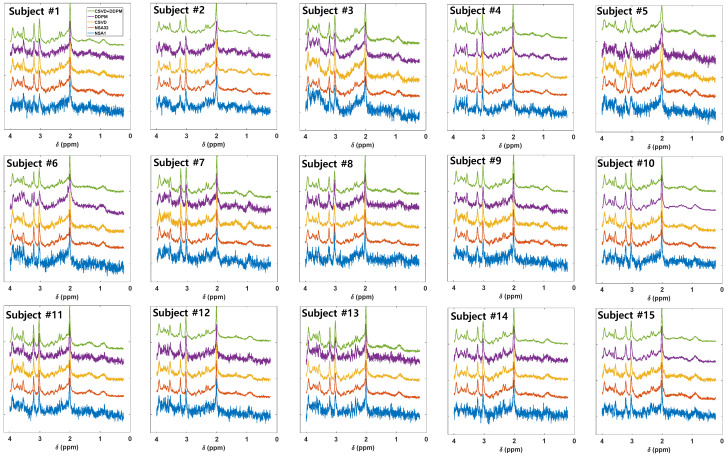
Comparison of methods for the baseline dataset (N = 15). Representative spectrum (1/32) in the baseline data for the following methods: NSA1 (no average, blue), NSA32 (average of 32 NSA1 scans, red), CSVD (denoising of NSA1 using CSVD, yellow), DDPM10 (denoising of NSA1 data using DDPM, violet), CSVD+DDPM2 (denoising of CSVD-denoised data using DDPM, green).

Figure 2 illustrates a visual inspection of the baseline dataset (N = 15) processed using different methods. The CSVD and CSVD+DDPM2 methods generated results with better spectral details and smaller noise than the NSA1 or DDPM10 methods, and the CSVD+DDPM2 method produced the highest SNR overall.

**Figure 3:**
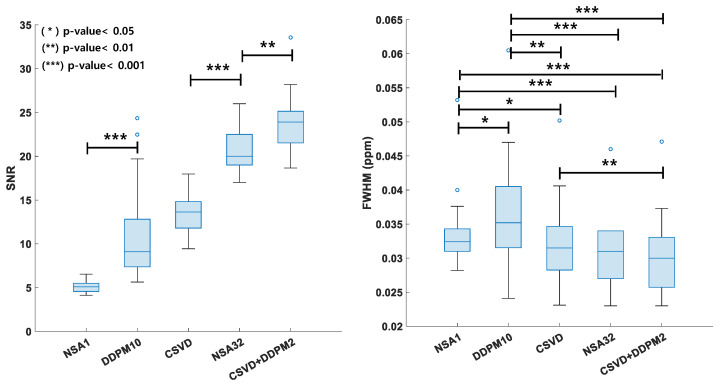
SNR (**left**) and FWHM (**right**) values (mean ± standard deviation) using different approaches, calculated from 15 baseline MRS datasets. Asterisks at the top of the boxplot indicate statistical significance: * *p*-value < 0.05, ** *p*-value < 0.01, *** *p*-value < 0.001. Methods include NSA1 (no signal average), DDPM10 (DDPM reverse denoising with 10 steps on NSA1 data), CSVD (Casorati singular value decomposition denoising on NSA1 data), NSA32 (32 signal averages), and CSVD+DDPM2 (DDPM reverse denoising with 2 steps on CSVD-denoised NSA1 data). SNR stands for signal-to-noise ratio, FWHM represents full width at half maximum. Significant differences were calculated using pairwise *t*-tests.

Figure 3 presents the SNR and FWHM values of the different methods on the baseline datasets, which show that the CSVD+DDPM2 methods have better quantification results than the other reference methods. The SNR values produced by NSA1, DDPM10, CSVD, NSA32, and CSVD+DDPM2 across all baseline datasets were 5.07 ± 0.81, 11.46 ± 6.51, 13.54 ± 3.13, 20.87 ± 2.45, and 23.97 ± 5.37, respectively. The results show that CSVD+DDPM2 outperforms NSA32 in terms of the SNR (*p* < 0.0001). The FWHM values produced by NSA1, DDPM10, CSVD, NSA32, and CSVD+DDPM2 across all baseline datasets were 0.0341 ± 0.0076, 0.0373 ± 0.0114, 0.0322 ± 0.0084, 0.0309 ± 0.0056, and 0.0306 ± 0.0074, respectively. The results show that CSVD+DDPM2 outperforms NSA32 in terms of SNR and is similar to the FWHM.

**Figure 4:**
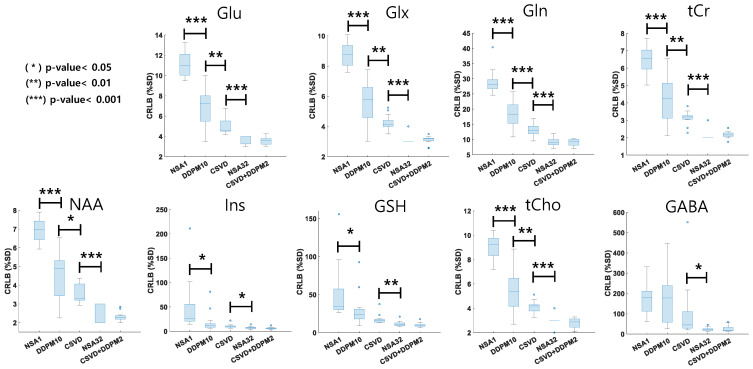
Comparisons of CRLB values from the baseline MRS dataset (mean ± standard deviation) across different denoising methods. Asterisks above the boxplot indicate statistical significance levels: * *p*-value < 0.05, ** *p*-value < 0.01, *** *p*-value < 0.001. Significant differences were assessed using pairwise *t*-tests. CRLB refers to the Cramer–Rao lower bound, which represents the standard error estimates returned by LCModel; lower CRLB values are associated with improved metabolite estimation.

Figure 4 illustrates a comparison of CRLB values for key metabolites across the different denoising methods. The results revealed that the denoising methods DDPM10, CSVD, and CSVD+DDPM2 consistently produced lower CRLB values compared to NSA1, approaching those observed with NSA32.

Specifically, the CRLB values obtained for Glu were 11.07 ± 1.24 for NSA1, 6.72 ± 1.93 for DDPM10, 4.96 ± 0.72 for CSVD, 3.73 ± 0.46 for NSA32, and 3.59 ± 0.40 for CSVD+DDPM2. For Glx, the CRLB values were 8.74 ± 0.75 for NSA1, 5.56 ± 1.48 for DDPM10, 4.23 ± 0.50 for CSVD, 3.07 ± 0.26 for NSA32, and 3.11 ± 0.25 for CSVD+DDPM2. In the case of Gln, the CRLB values were 29.00 ± 3.88 for NSA1, 18.01 ± 4.46 for DDPM10, 13.31 ± 2.01 for CSVD, 9.40 ± 1.55 for NSA32, and 9.06 ± 1.12 + CSVD+DDPM2. The CRLB values for tCr were 6.51 ± 0.74 for NSA1, 4.13 ± 1.30 for DDPM10, 3.14 ± 0.36 for CSVD, 2.20 ± 0.41 for NSA32, and 2.16 ± 0.18 for CSVD+DDPM2. For NAA, the CRLB values were 6.92 ± 0.63 for NSA1, 4.46 ± 1.34 for DDPM10, 3.55 ± 0.49 for CSVD, 2.40 ± 0.51 for NSA32, and 2.31 ± 0.23 for CSVD+DDPM2. Regarding Ins, the CRLB values were 49.42 ± 52.09 for NSA1, 18.46 ± 20.03 for DDPM10, 10.01 ± 3.84 for CSVD, 6.93 ± 2.91 for NSA32, and 6.09 ± 2.03 for CSVD+DDPM2. For GSH, the CRLB values were 51.27 ± 35.50 for NSA1, 28.54 ± 21.50 for DDPM10, 17.37 ± 6.10 for CSVD, 11.67 ± 3.42 for NSA32, and 10.03 ± 2.71 for CSVD+DDPM2. Finally, the CRLB values for tCho were 9.12 ± 0.95 for NSA1, 5.38 ± 1.67 for DDPM10, 4.12 ± 0.49 for CSVD, 3.00 ± 0.38 for NSA32, and 2.79 ± 0.39 for CSVD+DDPM2, while the values for GABA were 180.07 ± 79.90 for NSA1, 175.58 ± 129.90 for DDPM10, 109.13 ± 137.82 for CSVD, 23.20 ± 9.06 for NSA32, and 25.57 ± 15.15 for CSVD+DDPM2.

**Figure 5:**
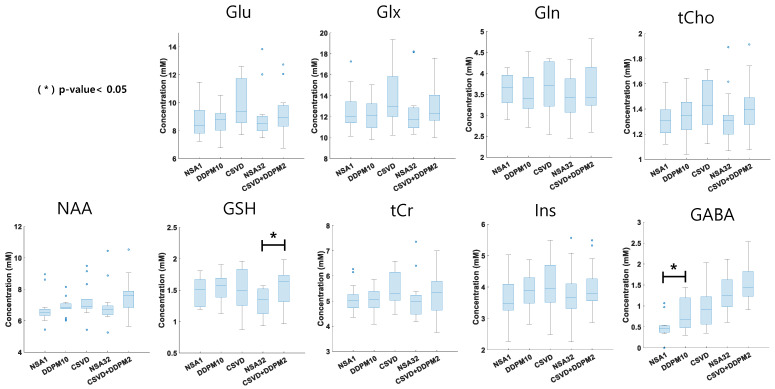
Comparisons of concentration values from the baseline MRS dataset (mean ± standard deviation) on different denoising methods. Asterisks at the top of the boxplot indicate statistical significance: * *p*-value < 0.05. Significant differences were calculated using pairwise *t*-tests.

Figure 5 shows a comparison of the LCModel-quantified metabolite concentrations across the different denoising methods based on the baseline dataset. Overall, there were no significant differences between the methods, except for GSH and GABA levels. Compared with NSA32, the CSVD+DDPM2 method overestimated GSH concentrations (*p* < 0.05). Additionally, NSA1 significantly underestimated GABA concentrations compared with DDPM10, CSVD, NSA32, and CSVD+DDPM2 (*p* < 0.05).

For Glu, the concentrations were 8.29 ± 2.61 for NSA1, 8.61 ± 1.00 for DDPM10, 9.72 ± 1.60 for CSVD, 8.94 ± 1.73 for NSA32, and 9.17 ± 1.57 for CSVD+DDPM2. Similarly, the concentrations of Glx were 11.82 ± 3.73 for NSA1, 12.15 ± 1.37 for DDPM10, 13.69 ± 2.67 for CSVD, 12.48 ± 2.47 for NSA32, and 12.91 ± 2.14 for CSVD+DDPM2.

In the case of Gln, the concentration values were 3.53 ± 1.18 for NSA1, 3.54 ± 0.51 for DDPM10, 3.97 ± 1.19 for CSVD, 3.54 ± 0.91 for NSA32, and 3.74 ± 0.75 for CSVD+DDPM2. tCr concentrations were 4.78 ± 1.43 for NSA1, 5.02 ± 0.52 for DDPM10, 5.39 ± 0.68 for CSVD, 5.07 ± 0.84 for NSA32, and 5.28 ± 0.81 for CSVD+DDPM2.

For NAA, the values were 6.29 ± 1.96 for NSA1, 6.87 ± 0.55 for DDPM10, 7.24 ± 1.07 for CSVD, 6.96 ± 1.22 for NSA32, and 7.49 ± 1.21 for CSVD+DDPM2. Concentrations of Ins were 3.49 ± 1.18 for the NSA1, 3.89 ± 0.57 for DDPM10, 3.99 ± 0.83 for CSVD, 3.75 ± 0.82 for NSA32, and 3.95 ± 0.78 for CSVD+DDPM2.

For GSH, the concentrations were 1.40 ± 0.44 for NSA1, 1.53 ± 0.24 for DDPM10, 1.46 ± 0.32 for CSVD, 1.32 ± 0.23 for NSA32, and 1.54 ± 0.28 for CSVD+DDPM2. Lastly, the concentrations of tCho were 1.25 ± 0.38 for NSA1, 1.34 ± 0.15 for DDPM10, 1.41 ± 0.19 for CSVD, 1.33 ± 0.21 for NSA32, and 1.41 ± 0.22 for CSVD+DDPM2, while the GABA concentrations were 0.49 ± 0.25 for NSA1, 0.79 ± 0.39 for DDPM10, 0.98 ± 0.53 for CSVD, 1.30 ± 0.46 for NSA32, and 1.44 ± 0.42 for CSVD+DDPM2.

### 3.2. Functional MRS Dataset

**Figure 6:**
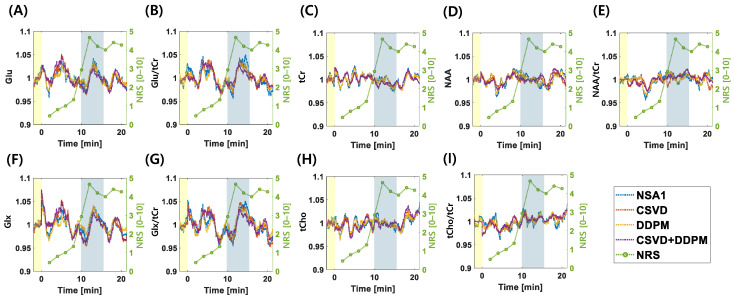
Changes in metabolite concentrations from average values in the functional MRS dataset (N = 13). Panels show (**A**) Glu, (**B**) Glu/tCr, (**C**) tCr, (**D**) NAA, (**E**) NAA/tCr, (**F**) Glx, (**G**) Glx/tCr, (**H**) tCho, and (**I**) tCho/tCr. Blue lines represent NSA1, red lines indicate CSVD, yellow lines show DDPM10, and violet lines illustrate CSVD+DDPM2. Green lines denote NRS (pain intensity ratings). Yellow shaded region represents the baseline period with no stimulation (Time < 0, duration = 3.12 min), while with capsaicin pain stimulation (Time > 0, duration = 22.4 min), the blue shaded region indicates the heat-activated period (duration = 4.4 min).

Figure 6 illustrates the average temporal changes in key metabolites quantified from the MRS datasets normalized to the mean values of the corresponding baseline and functional datasets. The horizontal axis features a yellow-shaded region prior to time point zero, representing the baseline period, and a blue-shaded region indicating the 4.4 min period during which heat was applied to the capsaicin-treated area of the forearm. Average subjective pain intensity ratings (NRSs) are shown in green on the right y-axis of each graph.

The analysis revealed differences in metabolite variability and correlations across various denoising methods. Glu variability: standard deviation from the mean: 0.0477 for NSA1, 0.0347 for DDPM10, 0.0357 for CSVD, and 0.0399 for CSVD+DDPM2. Kendall correlation values between Glu and NRS after 12 min: r = 0.4919, *p* < 0.001 for NSA1; r = 0.3842, *p* < 0.001 for DDPM10; r = 0.5740, *p* < 0.001 for CSVD; and r = 0.3861, *p* < 0.001 for CSVD+DDPM 2.

For Glx, the standard deviations from the mean were 0.0429 for NSA1, 0.0435 for DDPM10, 0.0352 for CSVD, and 0.0456 for CSVD+DDPM2. Kendall correlation values between Glx and NRS after 12 min: r = 0.2942, *p* < 0.001 for NSA1; r = 0.3565, *p* < 0.001 for DDPM10; r = 0.4612, *p* < 0.001 for CSVD; and r = 0.2837, *p* < 0.001 for CSVD+DDPM 2.

For Glu/tCr, the SDs from the mean were 0.0597 for NSA1, 0.0384 for DDPM10, 0.0430 for CSVD, and 0.0347 for CSVD+DDPM2. Kendall correlation values between Glu/tCr and NRS after 12 min: r = 0.1625, *p* < 0.01 NSA1; r = 0.1038, *p* = 0.0691 for DDPM10; r = 0.2597, *p* < 0.001 for CSVD; and r = 0.1879, *p* < 0.001 for CSVD+DDPM 2.

For Glx/tCr, the standard deviations from the mean were 0.0548 for NSA1, 0.0469 for DDPM10, 0.0428 for CSVD, and 0.0400 for CSVD+DDPM2. Kendall correlation values between Glx/tCr and NRS after 12 min: r = 0.0464, *p* = 0.4175 for NSA1; r = 0.1085, *p* = 0.0574 for DDPM10; r = 0.1442, *p* < 0.05 CSVD; r = 0.1702, *p* < 0.01; and CSVD+DDPM 2.

For tCr, the SDs from the mean were 0.0354 for NSA1, 0.0224 for DDPM10, 0.0288 for CSVD, and 0.0245 for CSVD+DDPM2. Kendall correlation values between tCr and NRS after 12 min: r = 0.1298, *p* < 0.05 NSA1; r = 0.0625, *p* = 0.2742 for DDPM10; r = 0.1970, *p* < 0.001 for CSVD; and r = 0.1749, *p* < 0.01 for CSVD+DDPM2.

For NAA, the standard deviations from the mean were 0.0337 for NSA1, 0.0239 for DDPM10, 0.0291 for CSVD, and 0.0292 for CSVD+DDPM2. Kendall correlation values between NAA and NRS after 12 min: r = 0.2597, *p* < 0.001 for NSA1; r = −0.0333, *p* = 0.5606 for DDPM10; r = −0.1800, *p* < 0.05 for CSVD; and r = −0.0767, *p* = 0.1795 for CSVD+DDPM2.

For tCho, the standard deviations from the mean were 0.0405 for NSA1, 0.0287 for DDPM10, 0.0320 for CSVD, and 0.0306 for CSVD+DDPM2. Kendall correlation values between tCho and NRS after 12 min: r = 0.1281, *p* < 0.05 for NSA1; r = −0.0245, *p* = 0.6694 for DDPM10; r = 0.1014, *p* = 0.0757 for CSVD; and r = −0.0925, *p* = 0.1053 for CSVD+DDPM2.

For NAA/tCr, the SDs from the mean were 0.0385 for NSA1, 0.0293 for DDPM10, 0.0313 for CSVD, and 0.0246 for CSVD+DDPM2. Kendall correlation values between NAA/tCr and NRS after 12 min: r = −0.0708, *p* = 0.2152 for NSA1; r = −0.2378, *p* < 0.001 for DDPM10; r = −0.3477, *p* < 0.001 for CSVD; and r = −0.1840, *p* < 0.01, for CSVD+DDPM 2.

For tCho/tCr, the SDs from the mean were 0.0479 for NSA1, 0.0236 for DDPM10, 0.0364 for CSVD, and 0.0215 for CSVD+DDPM2. Kendall correlation values between tCho/tCr and NRS after 12 min: r = −0.1717, *p* < 0.01 for NSA1; r = −0.1688, *p* < 0.01 for DDPM10; r = −0.1878, *p* < 0.01 for CSVD; and r = −0.1945, *p* < 0.001 for CSVD+DDPM 2.

**Figure 7:**
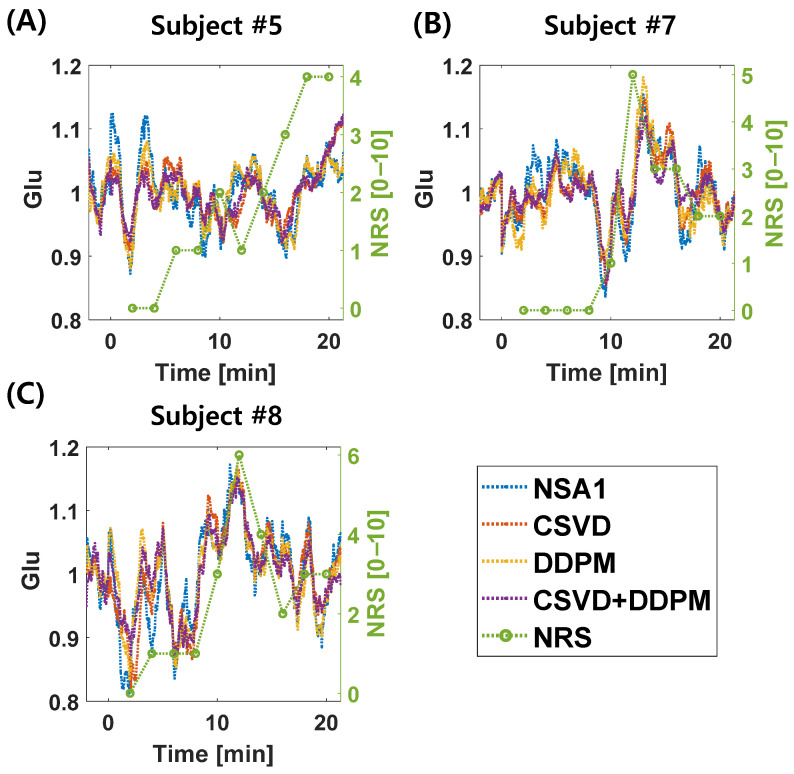
Changes in Glu concentrations from average values in the representative individual subjects. Panels show (**A**) subject #5, (**B**) subject #7, and (**C**) subject #8. Blue lines represent NSA1, red lines indicate CSVD, yellow lines show DDPM10, and violet lines illustrate CSVD+DDPM2. Green lines denote pain intensity rating (NRS) values.

Figure 7 shows the temporal changes in Glu levels in the four individual subjects. Although not all 15 subjects are shown, interestingly, similar patterns were observed between the changes in pain intensity ratings (NRS, shown in green) and Glu levels across the subjects.

For subject #5 (Figure 7A), the standard deviation from the mean was 0.0515 for NSA1, 0.0421 for DDPM10, 0.0434 for CSVD, and 0.0392 for CSVD+DDPM2. Kendall correlations between Glu changes and NRS after 12 min were as follows: NSA1 (r = 0.2115, *p* < 0.001), DDPM10 (r = 0.4192, *p* < 0.001), CSVD (r = 0.1683, *p* < 0.01), and CSVD+DDPM2 (r = 0.3437, *p* < 0.001).

For subject #7 (Figure 7B), the standard deviation from the mean was 0.0609 for NSA1, 0.0525 for DDPM10, 0.0586 for CSVD, and 0.0432 for CSVD+DDPM2. The Kendall correlations between Glu changes and NRS after 12 min were as follows: NSA1 (r = 0.4547, *p* < 0.001), DDPM10 (r = 0.4607, *p* < 0.001), CSVD (r = 0.5748, *p* < 0.001), and CSVD+DDPM2 (r = 0.5551, *p* < 0.001).

For subject #8 (Figure 7C), the standard deviation from the mean was 0.0776 for NSA1, 0.0735 for DDPM10, 0.0681 for CSVD, and 0.0620 for CSVD+DDPM2. The Kendall correlations between Glu changes and NRS after 12 min were as follows: NSA1 (r = 0.1445, *p* < 0.05), DDPM10 (r = 0.2754, *p* < 0.001), CSVD (r = 0.2926, *p* < 0.001), and CSVD+DDPM2 (r = 0.3656, *p* < 0.001).

## 4. Discussion

In this study, we proposed a hybrid denoising approach based on the CSVD and DDPM methods for ^1^H MRS data analysis and evaluated its performance on baseline and functional MRS datasets. Quantitative results show that the proposed DDPM-based frameworks with the CSVD denoising approach can achieve better performance than the CSVD and DDPM-only denoising and no averaging (e.g., NSA1), and are comparable to the traditional averaging approach (e.g., NSA32). We also performed functional MRS analysis in individual subjects, monitored dynamic changes in metabolites with high temporal resolution (e.g., TR = 4000 ms), and captured Glu level changes with pain stimulation tasks and other stable metabolites.

As shown in Figure 2 and Figure 3, CSVD and CSVD+DDPM2 methods outperformed DDPM10 and NSA1. The CSVD+DDPM2 approach demonstrated SNR improvements comparable to or even better than those achieved using NSA32. Additionally, there was no significant difference in the FWHM between NSA32 and CSVD+DDPM2, whereas the FWHM values for NSA1, DDPM10, and CSVD were all higher than those of NSA32 (Figure 3), highlighting the potential of the CSVD+DDPM2 method for high-quality denoising with minimal line broadening.

Although the CSVD method provided better denoising results than NSA1 and DDPM10, the SNR improvements were not as pronounced as those observed with NSA32 or CSVD+DDPM2 (Figure 3).

The DDPM10 denoising approach also improved the SNR compared to NSA1, yielding inconsistent results compared to CSVD or CSVD+DDPM2 (Figure 2 and Figure 3). For instance, while some subjects such as #10 and #15 in Figure 2 exhibited effective denoising, others such as #5 and #13 exhibited minimal improvement. Additionally, new peaks appeared near 2 ppm and 3.3 ppm in subject #6, suggesting that the DDPM10 approach may introduce artifacts in certain cases and that it is difficult to synthesize MRS data with high accuracy.

In contrast, the CSVD+DDPM2 method effectively eliminated the artifacts the new peaks generated by DDPM10 in subject #6, underscoring its robustness. One significant advantage of the DDPM-based framework over previous methods is its flexibility in adjusting the reverse denoising steps, allowing for it to be integrated with other denoising techniques. However, the application of the DDPM requires careful consideration. For instance, if too few reverse steps are used, the SNR may not improve sufficiently; if too many steps are used, artificial peaks may be introduced, or the spectrum may become over-smoothed and distorted. Therefore, careful tuning of the reverse denoising steps or consideration of a combined approach is advisable when utilizing the DDPM method.

As shown in Figure 4 and Figure 5, our evaluations indicated that the CSVD+DDPM2 method reduced CRLB values (Figure 4), similar to NSA32, while preserving the concentrations of major brain metabolites (Figure 5), except for GSH. This suggests that applying the CSVD+DDPM2 denoising method to the NSA1 data could yield results comparable to those of NSA32, offering the potential to significantly reduce MRS data acquisition by up to 1/32. This advantage might be especially valuable for the rapid assessment of emergency cases or for analyzing functional MRS data, making it a useful approach for clinical and neuroscience applications.

As part of our demonstration, we conducted spectral analysis for each TR in the functional MRS, as depicted in Figure 6 and Figure 7. This analysis of the individual spectra achieved a time resolution of 4 s, which is 30 times faster than that of the conventional method, which has a time resolution of 2 min [2]. Using this rapid analytical method, we observed temporal changes in key metabolites, confirming the results of previous studies [2]. Specifically, we found that levels of Glu, Glx, Glu/tCr, Glx/tCr, and tCr increased and were positively correlated with pain intensity ratings following pain onset (e.g., NRS score ≥ 2, approximately after 12 min in our analysis). No significant changes in NRS were observed for other metabolites such as NAA and tCho. Additionally, the negative correlations observed between NAA/tCr and tCho/tCr could reflect increased tCr levels with pain stimulation.

Notably, without denoising (i.e., NSA1), inconsistent results were observed, such as a positive correlation between NAA and NRS, no correlation between Glx/tCr and NRS, and a positive correlation between tCho and NRS. Conversely, CSVD+DDPM2 produced consistent results, showing positive correlations with neurotransmitter levels, and no correlations with other metabolites. This is consistent with a previous study [2], demonstrating the potential benefits of the denoising process.

Using this approach, as illustrated in Figure 7, it may be possible to perform functional MRS data analysis on an individual basis. In certain subjects (e.g., subjects #5, #7, and #8 in Figure 7), changes in Glu levels closely aligned with changes in pain intensity ratings, showing a similar pattern. Although all methods (NSA1, CSVD, DDPM10, and CSVD+DDPM2) exhibited this pattern, compared with NSA1, the application of denoising techniques led to a reduction in variability and showed strong positive correlations. These findings suggest the potential of using functional MRS in event-related task studies [3] at the individual level to enhance the ability to analyze subject-specific responses in greater detail.

Furthermore, we obtained intriguing results using the proposed analysis method. While a previous study [2] reported that Glu and Glx levels transiently increased after pain onset, our analysis revealed that Glu and Glx levels fluctuated throughout the stimulation period, starting with the application of capsaicin to the forearm (Figure 6 and Figure 7). We suggest that the participants experienced continuous pain throughout the stimulation period. This fluctuation pattern was consistent across all methods (NSA1, DDPM10, CSVD, and CSVD+DDPM2). The discrepancies with previous findings might be attributed to differences in the time resolution, which could have masked these changes in an earlier study.

Our study has some limitations: First, owing to the small sample size (N = 15), the performance and validation results of the denoising models, such as CSVD and DDPM, may not be fully reliable. Future studies should include additional samples to address this limitation. Moreover, in the CSVD denoising process, the rank and lambda parameters are adjusted based on empirical tuning. Incorporating automatic selection methods such as optimal singular value hard thresholding (SVHT) or Marchenko–Pastur (MP) distribution-based approaches can improve processing algorithm [11,12,23]. Furthermore, we found that using the DDPM model alone, without the assistance of CSVD, made it challenging to effectively remove noise or risk over-denoising and subsequent distortion of the spectrum, resulting in unreliable quantification. A potential solution to avoid this issue could be to adopt a self-supervised denoising approach [21], such as CSVD denoising, which exclusively uses individual subject data. Finally, although our proposed method demonstrated a reduction in the quantification uncertainty for key metabolites, such as NAA, Glu, and tCr, further validation with a more diverse dataset is needed to confirm the effectiveness of our approach, especially for metabolites such as Ins, GSH, and GABA, which are not easily visible in the spectrum.

Future studies can explore the following directions: We considered methods to further reduce scan times. One potential approach is to eliminate the water suppression period during MRS data acquisition. This technique, known as non-water-suppressed MRS [24], involves the simultaneous measurement of both metabolite and water signals, which can significantly shorten the scan time (e.g., ~500 ms per TR). However, the accurate extraction of the water signal (water removal) is complex and challenging, often resulting in residual sideband artifacts even after water removal [23,24]. We suggest that the DDPM may be a useful approach for mitigating these artifacts. By combining water removal techniques (e.g., CSVD) [23,24] and artifact removal methods using the DDPM, it may be possible to effectively reconstruct water-removed MRS data.

Additionally, the proposed method can be extended to various MRS(I) applications such as edited MRS [25,26,27], studies on various diseases [28,29], different functional MRS tasks [3], and other types of X-nuclei MRS such as ^31^P MRS [8,13,16], ^13^C MRS [9], and ^2^H echo-planar spectroscopic imaging [30]. Exploring these possibilities could enhance the utility and versatility of MRS in various research contexts.

## 5. Conclusions

We introduced a hybrid denoising method combining CSVD and DDPM for ^1^H MRS, which demonstrated improved performance over individual denoising techniques and NSA1. The CSVD+DDPM 2 approach effectively enhanced the SNR and maintained the spectral quality, allowing for high-resolution temporal analysis of metabolite changes. Despite these promising results, further validation with larger datasets is required to confirm the effectiveness of this method for all metabolites. Future work should explore reducing the scan time and applying the proposed method to various MRS types and research contexts.

## Data Availability

MRS data are openly available at (https://www.nitrc.org/projects/fmrs_2020/ (accessed on 13 May 2024)).

## Supplementary Materials

The following supporting information can be downloaded from https://www.mdpi.com/article/10.3390/bioengineering11111170/s1. Figure S1: CSVD denoising results of baseline ^1^H MRS data are shown with a gradually increasing rank parameter r and a fixed regularization parameter λ=500 (yellow lines). The blue lines represent the input noisy baseline ^1^H MRS data (NSA1), while the orange lines denote the reference data (NSA32). Denoising performance was assessed using PSNR and RMSE metrics, with the red box highlighting the case of maximum PSNR and minimum RMSE. CSVD: Casorati singular value decomposition; NSA: number of signal averages; PSNR: peak signal-to-noise ratio; RMSE: root mean square error. Figure S2: CSVD denoising results of baseline ^1^H MRS data are shown with a gradually increasing regularization parameter λ and a fixed rank parameter r = 2 (yellow lines). The blue lines represent the input noisy baseline ^1^H MRS data (NSA1), while the orange lines denote the reference data (NSA32). Denoising performance was assessed using PSNR and RMSE metrics, with the red box highlighting the case of maximum PSNR and minimum RMSE. CSVD: Casorati singular value decomposition; NSA: number of signal averages; PSNR: peak signal-to-noise ratio; RMSE: root mean square error. Figure S3: PSNR and normalized singular value of baseline and functional ^1^H MRS data. (A) PSNR of baseline 1H MRS data at various thresholds r and regularization parameter λ. (B) Normalized singular value of baseline 1H MRS data. (C) PSNR of functional ^1^H MRS data at different thresholds r. (D) Normalized singular value of functional ^1^H MRS data. PSNR: peak signal-to-noise ratio. Figure S4: CSVD denoising results using a lasso penalty on baseline ^1^H MRS data are shown across various combinations of the ℓ1-norm regularization parameter λ, rank r, augmented Lagrangian parameter ρ, with iterations fixed at 100 (yellow lines). The blue lines represent the noisy baseline ^1^H MRS data (NSA1), and the orange lines indicate the reference data (NSA32). Denoising performance was evaluated using PSNR and RMSE metrics. The red box highlights the case with maximum PSNR and minimum RMSE. CSVD: Casorati singular value decomposition; NSA: number of signal averages; PSNR: peak signal-to-noise ratio; RMSE: root mean square error. Figure S5: DDPM denoising results of baseline ^1^H MRS data are shown with a gradually increasing reverse denoising steps (violet lines). The blue lines represent the input noisy baseline ^1^H MRS data (NSA1), while the orange lines denote the reference data (NSA32). Denoising performance was assessed using PSNR and RMSE metrics, with the red box highlighting the case of maximum PSNR and minimum RMSE. DDPM: denoising diffusion probabilistic model; NSA: number of signal averages; PSNR: peak signal-to-noise ratio; RMSE: root mean square error. Figure S6: CSVD+DDPM denoising results of baseline ^1^H MRS data are shown with a gradually increasing reverse denoising steps (green lines). The blue lines represent the input noisy baseline ^1^H MRS data (NSA1), while the orange lines denote the reference data (NSA32). Denoising performance was assessed using PSNR and RMSE metrics, with the red box highlighting the case of maximum PSNR and minimum RMSE. CSVD+DDPM: hybrid denoising model; NSA: number of signal averages; PSNR: peak signal-to-noise ratio; RMSE: root mean square error. Figure S7: PSNR and RMSE. (A) PSNR at various number of reverse denoising steps using DDPM-only approach. (B) RMSE at various number of reverse denoising steps using DDPM-only approach. (C) PSNR at various reverse denoising steps using CSVD+DDPM approach. (D) RMSE at various reverse denoising steps using CSVD+DDPM approach. CSVD+DDPM: hybrid denoising model; PSNR: peak signal-to-noise ratio; RMSE: root mean square error. Figure S8: Denoising results of baseline 1H MRS data are shown for the (A) CSVD+DDPM (green lines), (B) DnCNN (cyan lines), and (C) CSVD+DnCNN (magenta lines) models. The blue lines represent the input noisy baseline ^1^H MRS data (NSA1), while the orange lines denote the reference data (NSA32). Denoising performance was assessed using PSNR and RMSE metrics. The spectral distortions are indicated by yellow arrows. CSVD+DDPM: hybrid denoising model; NSA: number of signal averages; PSNR: peak signal-to-noise ratio; RMSE: root mean square error. DnCNN: denoising convolution neural network; CSVD+DnCNN: hybrid denoising model with DnCNN. Figure S9: Training learning curve for a DDPM model, with zoomed-in areas highlighted in a red box. Figure S10: Glu concentration changes from average values in the functional MRS data (subject #1). (A) Glu changes with NSA1 (blue), CSVD (orange), DDPM20 (yellow), and CSVD+DDPM5 (violet) approaches. (B) Glu changes with NSA1 (blue), CSVD (orange), DDPM10 (yellow), and CSVD+DDPM2 (violet) approaches. Red arrows and circles indicate incorrect LCModel quantifications that may be caused by spectrum distortions from the DDPM model. CSVD+DDPM: hybrid denoising model; NSA: number of signal averages; Glu: glutamate; NRS: pain intensity rating; DDPM: denoising diffusion probabilistic model; CSVD: Casorati singular value decomposition. Figure S11: Glu concentration changes from average values in the functional MRS data. Glu changes with NSA1 (blue), CSVD (orange), DDPM10 (yellow), and CSVD+DDPM2 (violet) approaches are shown. Red arrows in subject #14 and subject #15 indicate incorrect quantifications or artifacts.

## Appendix A

### Appendix A.1. Derivation of the Closed-Form Solution for CSVD Denoising

The goal is to minimize the following cost function:

(A1) $$J\;=\;{\left\|\hat{s}\;-s\right\|}_{2}^{2}+{\lambda \left\|U_{r}U_{r}^{*}\hat{s}\;-\hat{s}\right\|}_{2}^{2}$$

Which can be rewritten as

(A2) $$J\;=\;{\left\|I\hat{s}\;-s\right\|}_{2}^{2}+{\left\|\sqrt{\lambda }(U_{r}U_{r}^{*}\;-I)\hat{s}\right\|}_{2}^{2}$$

The cost function can be expressed more compactly using block matrix and vector notation:

(A3) $$J={\left\|\left[\begin{array}{c} I\hat{s}-s\\ \sqrt{\lambda }\;\left(U_{r}U_{r}^{*}\;-I\right)\hat{s} \end{array}\right]\right\|}_{2}^{2}$$

This can be further simplified to

(A4) $$J\;=\;{\left\|\left[\begin{array}{c} I\\ \sqrt{\lambda }(U_{r}U_{r}^{*}\;-I) \end{array}\right]\hat{s}-\left[\begin{array}{c} s\\ 0 \end{array}\right]\right\|}_{2}^{2}\;=\;{\left\|A\hat{s}-b\right\|}_{2}^{2}$$

where

(A5) $$A=\left[\begin{array}{c} I\\ \sqrt{\lambda }(U_{r}U_{r}^{*}\;-I) \end{array}\right],\;b=\;\left[\begin{array}{c} s\\ 0 \end{array}\right]$$

Expanding the cost function:

(A6) $$J={\left(A\hat{s}-b\right)}^{*}\left(A\;\hat{s}-b\right)$$

To find the minimizer $\hat{s}$, we differentiate J with respect to $\hat{s}$ and set the derivative to zero.

(A7) $$\frac{\partial J}{\partial \hat{s}}=2A^{*}A\hat{s}-2A^{*}b=0$$

Simplifying this equation gives

(A8) $$A^{*}A\hat{s}=A^{*}b$$

Substituting the expressions for A and b:

(A9) $$\left[\begin{array}{cc} I & \sqrt{\lambda }{\left(U_{r}U_{r}^{*}\;-I\right)}^{*}\; \end{array}\right]\;\left[\begin{array}{c} I\\ \sqrt{\lambda }(U_{r}U_{r}^{*}\;-I) \end{array}\right]\hat{s}\;=\;\left[\begin{array}{cc} I & \sqrt{\lambda }{\left(U_{r}U_{r}^{*}\;-I\right)}^{*} \end{array}\right]\left[\begin{array}{c} s\\ 0 \end{array}\right]$$

This simplifies to

(A10) $$(I+\lambda {\left(U_{r}U_{r}^{*}-I\right)}^{*}\left(U_{r}U_{r}^{*}-I\right))\;\hat{s}=s$$

Solving for $\hat{s}$:

(A11) $$\hat{s}={\left(I+\lambda {\left(U_{r}U_{r}^{*}-I\right)}^{*}\left(U_{r}U_{r}^{*}-I\right)\right)}^{-1}s$$

where * is the conjugate transpose and I is an identity matrix. This is the closed-form solution for $\hat{s}$, as denoted by Equation (2) in the problem statement.
