# Supplementary material for: Improving Brain Metabolite Detection with a Combined Low-Rank Approximation and Denoising Diffusion Probabilistic Model Approach
Source: Bioengineering (Basel). 2024 Nov 20;11(11):1170. doi: 10.3390/bioengineering11111170 (PMC11592133; doi:10.3390/bioengineering11111170)
Supplement: Supplementary file 1 [file bioengineering-11-01170-s001.zip › bioengineering-3288730-supplementary.pdf]

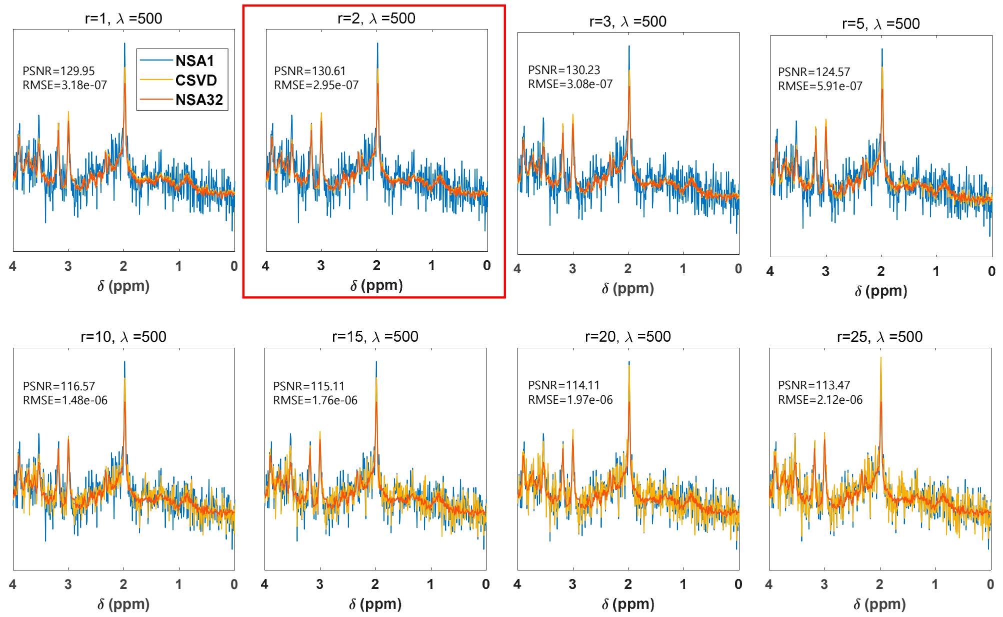

Figure S1. CSVD denoising results of baseline ^{1}H MRS data are shown with a gradually increasing rank parameter r and a fixed regularization parameter $\lambda = 500$ (yellow lines). The blue lines represent the input noisy baseline ^{1}H MRS data (NSA1), while the orange lines denote the reference data (NSA32). Denoising performance was assessed using PSNR and RMSE metrics, with the red box highlighting the case of maximum PSNR and minimum RMSE. **CSVD**, Casorati singular value decomposition; **NSA**, number of signal averages, **PSNR**, peak signal-to-noise ratio, **RMSE**, root mean square error.

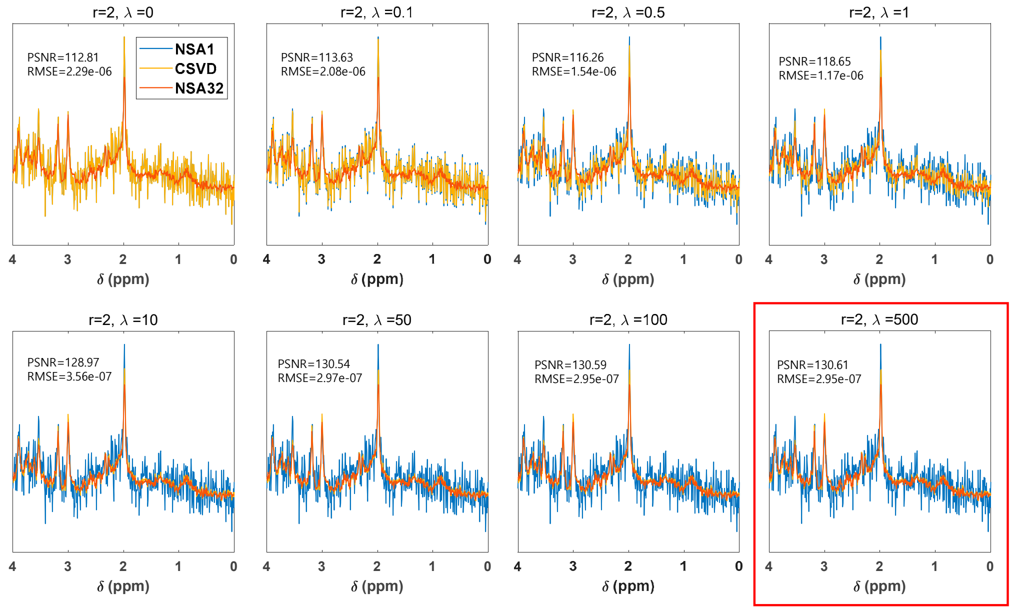

Figure S2. CSVD denoising results of baseline ^{1}H MRS data are shown with a gradually increasing regularization parameter λ and a fixed rank parameter r=2 (yellow lines). The blue lines represent the input noisy baseline ^{1}H MRS data (NSA1), while the orange lines denote the reference data (NSA32). Denoising performance was assessed using PSNR and RMSE metrics, with the red box highlighting the case of maximum PSNR and minimum RMSE. **CSVD**, Casorati singular value decomposition; **NSA**, number of signal averages, **PSNR**, peak signal-to-noise ratio, **RMSE**, root mean square error.

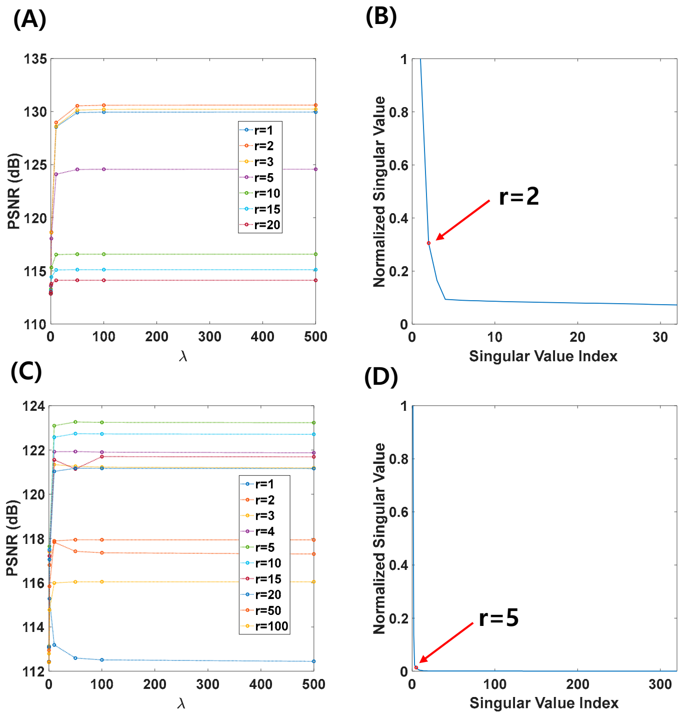

Figure S3. PSNR and normalized singular value of baseline and functional ^{1}H MRS data. (A) PSNR of baseline ^{1}H MRS data at various thresholds r and regularization parameter λ. (B) Normalized singular value of baseline ^{1}H MRS data. (C) PSNR of functional ^{1}H MRS data at different thresholds r. (D) Normalized singular value of functional ^{1}H MRS data. **PSNR**, peak signal-to-noise ratio.

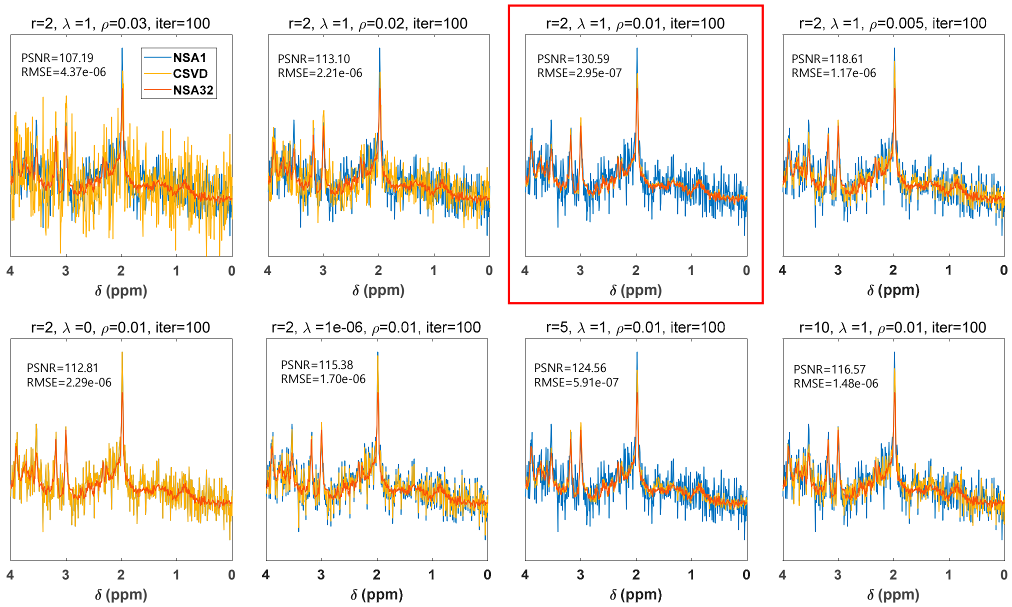

Figure S4. CSVD denoising results using a lasso penalty on baseline ^{1}H MRS data are shown across various combinations of the ℓ1-norm regularization parameter λ, rank r, augmented Lagrangian parameter ρ, with iterations fixed at 100 (yellow lines). The blue lines represent the noisy baseline ^{1}H MRS data (NSA1), and the orange lines indicate the reference data (NSA32). Denoising performance was evaluated using PSNR and RMSE metrics. The red box highlights the case with maximum PSNR and minimum RMSE. **CSVD**, Casorati singular value decomposition; **NSA**, number of signal averages, **PSNR**, peak signal-to-noise ratio, **RMSE**, root mean square error.

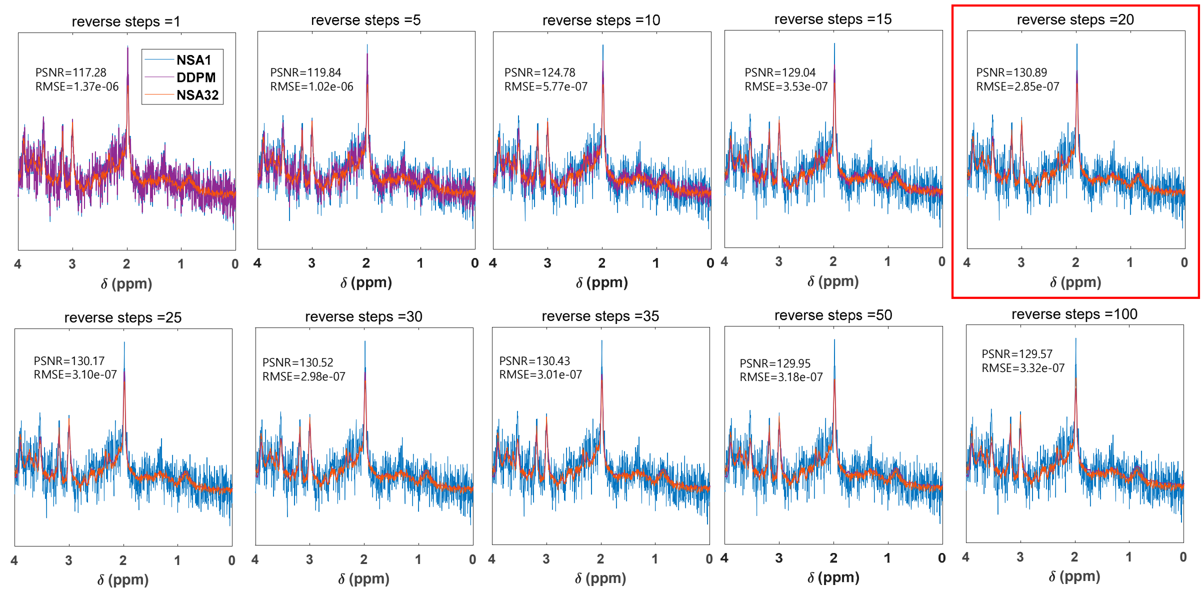

Figure S5. DDPM denoising results of baseline ^{1}H MRS data are shown with a gradually increasing reverse denoising steps (violet lines). The blue lines represent the input noisy baseline ^{1}H MRS data (NSA1), while the orange lines denote the reference data (NSA32). Denoising performance was assessed using PSNR and RMSE metrics, with the red box highlighting the case of maximum PSNR and minimum RMSE. **DDPM**, denoising diffusion probabilistic model; **NSA**, number of signal averages, **PSNR**, peak signal-to-noise ratio, **RMSE**, root mean square error.

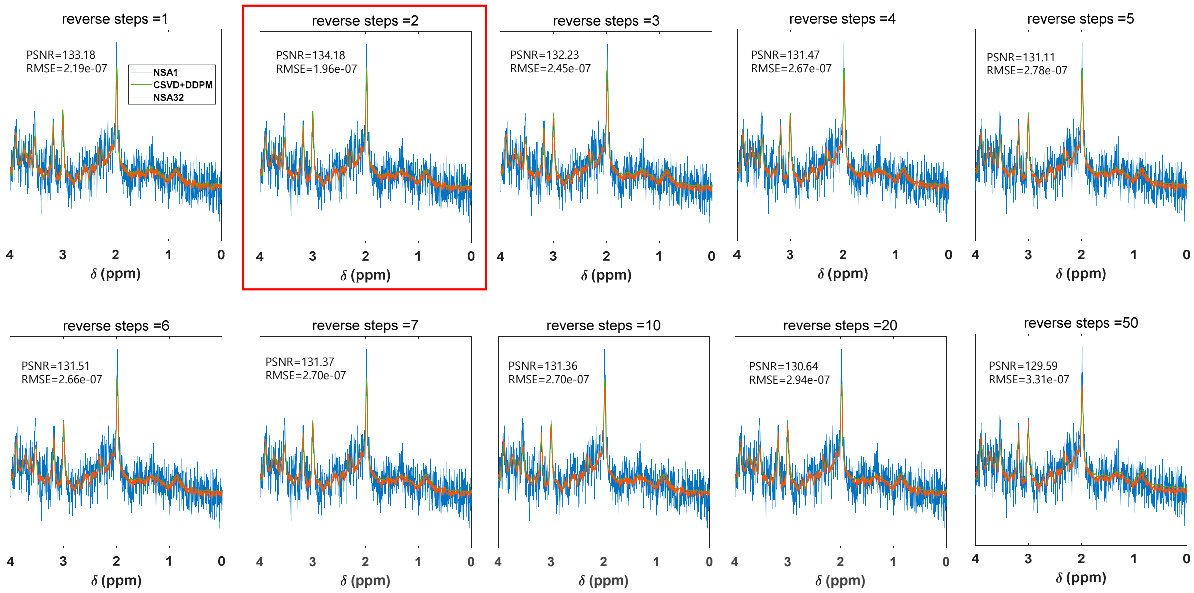

Figure S6. CSVD+DDPM denoising results of baseline ^{1}H MRS data are shown with a gradually increasing reverse denoising steps (green lines). The blue lines represent the input noisy baseline ^{1}H MRS data (NSA1), while the orange lines denote the reference data (NSA32). Denoising performance was assessed using PSNR and RMSE metrics, with the red box highlighting the case of maximum PSNR and minimum RMSE. **CSVD+DDPM**, hybrid denoising model; **NSA**, number of signal averages, **PSNR**, peak signal-to-noise ratio, **RMSE**, root mean square error.

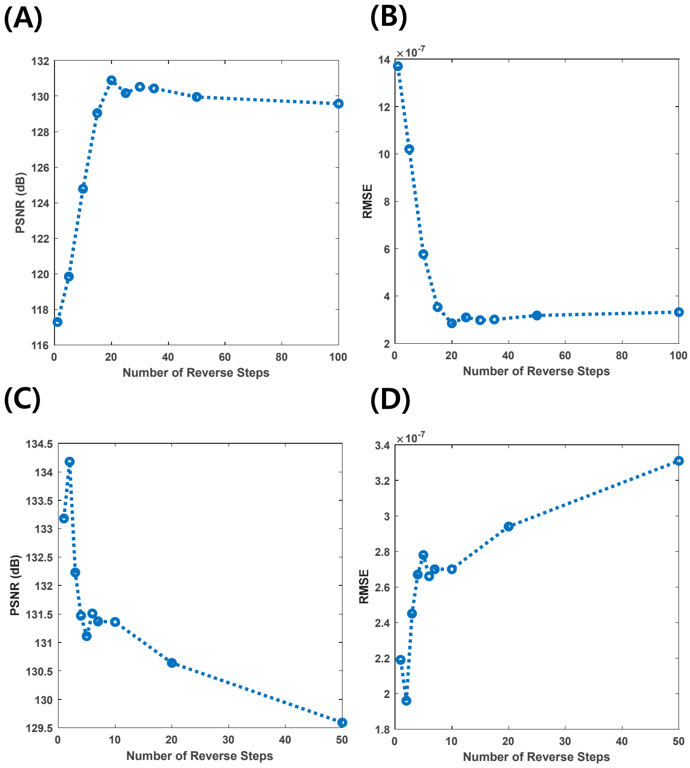

Figure S7. PSNR and RMSE. (A) PSNR at various number of reverse denoising steps using DDPM-only approach. (B) RMSE at various number of reverse denoising steps using DDPM-only approach. (C) PSNR at various reverse denoising steps using CSVD+DDPM approach. (D) RMSE at various reverse denoising steps using CSVD+DDPM approach. **CSVD+DDPM**, hybrid denoising model; **PSNR**, peak signal-to-noise ratio, **RMSE**, root mean square error.

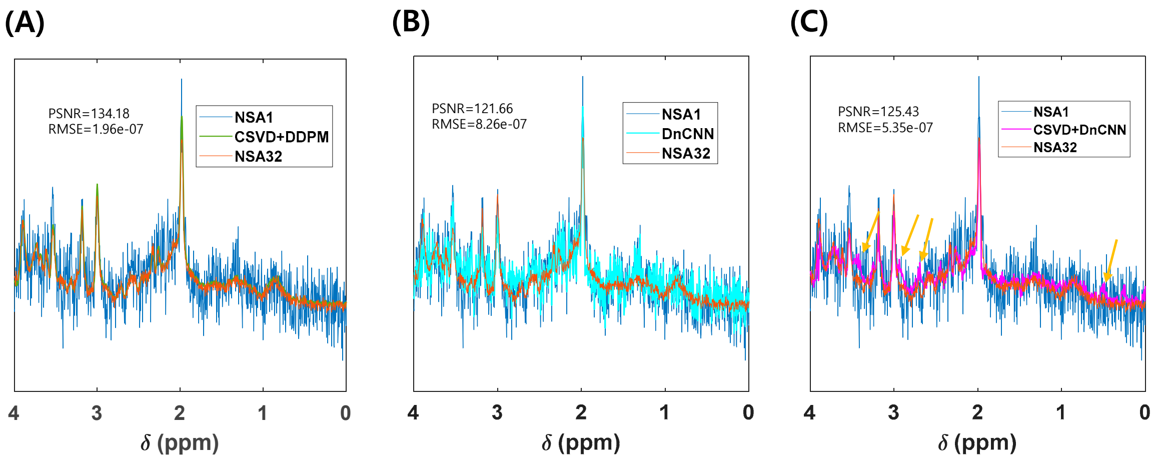

Figure S8. Denoising results of baseline ^{1}H MRS data are shown for the (A) CSVD+DDPM (green lines), (B) DnCNN (cyan lines) and (C) CSVD+DnCNN (magenta lines) models. The blue lines represent the input noisy baseline ^{1}H MRS data (NSA1), while the orange lines denote the reference data (NSA32). Denoising performance was assessed using PSNR and RMSE metrics. The spectral distortions are indicated by yellow arrows. **CSVD+DDPM**, hybrid denoising model; **NSA**, number of signal averages, **PSNR**, peak signal-to-noise ratio, **RMSE**, root mean square error. DnCNN, denoising convolution neural network, CSVD+DnCNN, hybrid denoising model with DnCNN.

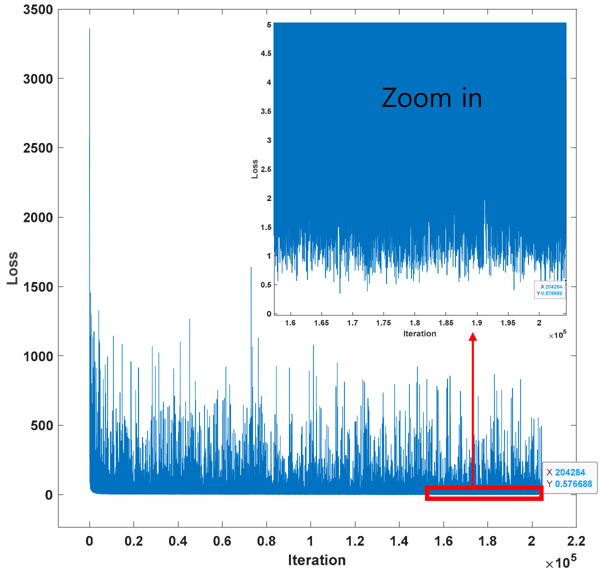

Figure S9. Training learning curve for a DDPM model, with zoomed-in areas highlighted in a red box.

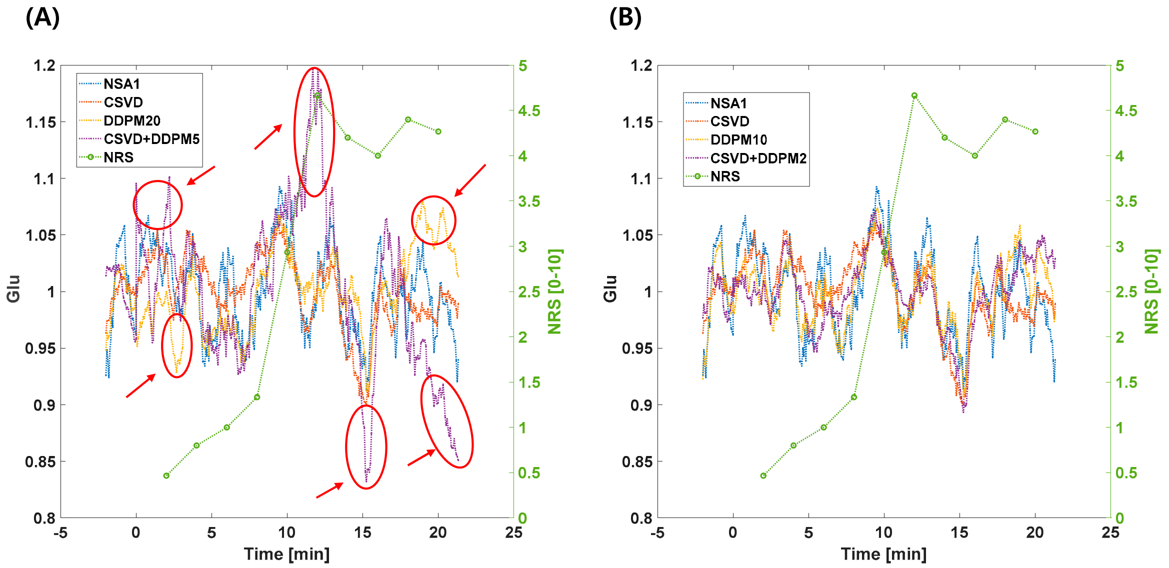

Figure S10. Glu concentration changes from average values in the functional MRS data (subject #1). (A) Glu changes with NSA1 (blue), CSVD (orange), DDPM20 (yellow), and CSVD+DDPM5 (violet) approaches. (B) Glu changes with NSA1 (blue), CSVD (orange), DDPM10 (yellow), and CSVD+DDPM2 (violet) approaches. Red arrows and circles indicate incorrect LCModel quantifications that may be caused by spectrum distortions from the DDPM model. **CSVD+DDPM**, hybrid denoising model; **NSA**, number of signal averages, **Glu**, glutamate, **NRS**, pain intensity rating; **DDPM**, denoising diffusion probabilistic model; **CSVD**, casorati singular value decomposition.

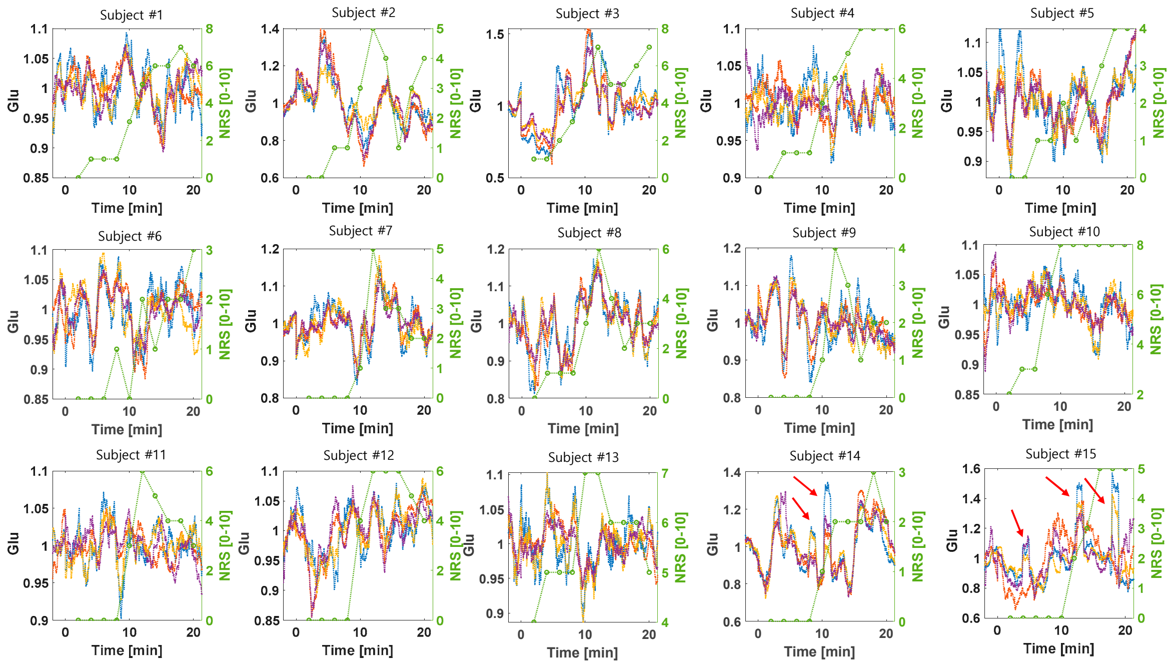

Figure S11. Glu concentration changes from average values in the functional MRS data. Glu changes with NSA1 (blue), CSVD (orange), DDPM10 (yellow), and CSVD+DDPM2 (violet) approaches are shown. Red arrows in subject #14 and subject #15 indicate incorrect quantifications or artifacts.
